# Supplementary material for: Asthma clinical decision support systems in primary care: an updated scoping review of implementation
Source: NPJ Prim Care Respir Med. 2026 Mar 26;36:31. doi: 10.1038/s41533-026-00498-2 (PMC13187283; doi:10.1038/s41533-026-00498-2)
Supplement: Supplementary file 1 — Supplementary Information [file 41533_2026_498_MOESM1_ESM.docx]

Asthma Clinical Decision Support Systems in Primary Care: An Updated Scoping Review of Implementation

**Authors:**

Holly Tibble [0000-0001-7169-4087] ^1^,

Bohee Lee [0000-0002-9671-386X] ^2^,

Imogen Skene [0000-0001-5215-2899] ^3,4^

**Affiliations:**

1. Usher Institute, University of Edinburgh, UK
2. National Heart and Lung Institute, Imperial College London, UK
3. Barts Health NHS Trust, London, UK
4. Wolfson Institute of Population Health, Queen Mary University of London, UK

**Correspondence:**

Correspondence to Holly Tibble ([htibble@ed.ac.uk](mailto:htibble@ed.ac.uk))

**Author Contributions:**

HT conceived the scoping review was the lead author of the manuscript. HT, BL, and IS all screened the identified publications and extracted data. HT conducted data synthesis and analysis. All authors approved the analysis plan, reviewed and approved the final manuscript.

**Acknowledgements:** None

**Funding:** None Declared

**Competing Interests:**

All authors declare no financial or non-financial competing interests.

# Abstract

Introduction:

Despite advances in digital health technologies, the integration of clinical decision support systems (CDSSs) into routine primary care for asthma has been extremely limited. Asthma CDSSs employ diverse approaches, from prescribing support to risk prediction, but it remains unclear which are most likely to achieve sustained, real-world impact. Our objectives were to determine the mechanisms of action, clinical practice integration approaches, outputs and outcomes of asthma CDSSs in recent literature.

Methods:

Five electronic databases (Embase via Ovid, PubMed, CENTRAL, the Health Technology Assessment (HTA) Database, and the ISRCTN registry of clinical trials) were searched to identify papers published between 2012 and 2024 describing pilot studies, feasibility studies, or clinical trials, of primary care-based asthma CDSS. Two independent reviewers screened the retrieved literature and extracted the data on study designs, interventions, outcomes and mechanisms of action, and results.

Results:

Across 18 included trials, interventions demonstrated substantial heterogeneity in mechanisms, integration methods, and targeted clinical behaviours. Although some studies showed improvements in adherence to prescribing best practices and the delivery of personalised action plans, most reported modest or declining system use over time and inconsistent effects on asthma control or severe attack outcomes.

Conclusion:

Continued progress will depend on integrating behavioural theory, improving workflow compatibility, and generating rigorous evidence to guide the development of CDSSs with genuine potential for sustainable impact.

# Key Messages

What is already known on this topic:

A 2014 systematic review of asthma CDSS trials highlighted that low system uptake was a substantial barrier to improvements in patient outcomes, and stipulated that it was essential that future research focussed on developing interventions that were aligned with existing clinical workflows.

What this study adds:

In the last decade, advances in digital health technology have resulted in an increase in asthma CDSS trials. However, issues with uptake and sustainability have persisted.

How this study might affect research, practice or policy:

This review highlights the need for theory informed intervention development, clearly defined proposed mechanisms of action, and mixed-methods trial designs, in order to improve clinician support and engagement.

# Introduction

Asthma attacks are the cause of more than 25 deaths per week on average in the United Kingdom (UK) ^1,2^. Primary care consultations provide the opportunity for patients and clinicians to assess fluctuations in asthma attack risk. Accurate prediction of asthma attack risk can instigate timely primary care intervention, prompt well-timed primary care visits, promote risk-reducing lifestyle choices, and encourage patients to seek emergency care following symptom deterioration. Furthermore, highlighting periods when risk is lower can reduce lifetime steroid use and patient anxiety.

Clinical Decision Support Systems (CDSSs) are digital tools which can be integrated into clinical practice to assist diagnosis, prognosis, treatment, and clinical processes such as prompting follow-up and triaging patients ^3^. These can be driven by data-driven statistical prediction models, or expert-knowledge-based rulesets and processes.

There have been successful intervention trials predicting asthma attack risks in primary care including in spirometry test result support ^4^, flagging high-risk patients in primary care ^5^, identifying those overdue for influenza vaccination ^6^ (respiratory infections are a common trigger for asthma symptom deterioration), and improving quality of care by adherence to evidence-based guidelines ^7–10^. Despite some promising results, there are no CDSS currently endorsed for clinicians by either the Global Initiative for Asthma guidelines (GINA; 2020) ^11^ or the joint guidelines by the British Thoracic Society and Scottish Intercollegiate Guidelines Network (BTS/SIGN; 2019) ^12^, and are not referenced at all in the 2020 National Institute of Clinical Excellence (NICE) guidelines ^13^. This lack of integration into routine clinical practice is not isolated to asthma: indeed very few CDSSs have had sustainable success beyond (or even within) initial implementation trials ^14^. While many of the aforementioned trials were able to demonstrate good model performance with testing data, positive impact on behaviour change has been more difficult to demonstrate in implementation studies.

Understanding what makes a CDSS successful is of great value to researchers and developers. Features such as requiring a reason entered for over-riding the system’s advice and presenting the output of the model as both advice to the practitioner and to the patient may promote behaviour change in users ^15^. Transparency regarding the capabilities and limitations of the system, including known population subgroups with lower accuracy and the diversity of the training data, is extremely valuable to help clinicians decide whether to override their own judgement ^16^. Some clinicians particularly liked to be able to use a demonstration tool with patients, and so appreciated clear graphics and the option for a print-out to be generated ^17^. Beyond the initial trial, continued use of a CDSS relies on sustainable behaviour change, in the absence of incentives and monitoring ^18,19^.

In 2014, Matui *et al.* conducted a systematic review of Computer decision support systems trials for asthma between 1990 and 2012 ^20^. They identified eight trials in this period, but highlighted that the systems were commonly affected by low system uptake. They concluded that the ‘current generation’ of CDSSs were ‘unlikely to result in improvements in outcomes for patients with asthma’, and that future work was needed to better align systems with professional workflows. Substantial advances in digital health in the last decade underscore the value of reviewing developments that may improve asthma care.

Since the publication of the 2014 review by Matui *et al.* ^20^, and in particular after the start of the Covid-19 pandemic, the digital health landscape has undergone considerable transformation. There have been major advances in data infrastructure, interoperability, machine learning methods, and the integration of electronic health records into routine care within the UK. These developments have enabled the creation of more sophisticated, user-centred clinical decision support tools and have prompted renewed efforts to embed decision support within primary care workflows. Given these changes, and the ongoing clinical need for effective asthma risk-prediction and management tools, an updated review is warranted to assess whether recent CDSS interventions demonstrate improved uptake, alignment with clinical practice, and measurable benefits for patients.

The aim of this study was to identify asthma CDSS implementation trials, and review their design, outcomes, and effectiveness for feasibility and benefit of widescale implementation. Our objectives were to review the mechanisms of action, clinical practice integration approaches, outputs and outcomes of asthma CDSSs in recent literature.

# Methods

## Review Design and Methodological Framework

The protocol for this review is published in the Open Science Framework (OSF) repository ^21^. We conducted the review following the Preferred Reporting Items for Systematic reviews and Meta-Analyses (PRISMA) scoping review extension (PRISMA-ScR) methodology for undertaking and reporting scoping reviews ^22^, as reported in Appendix D.

This scoping review followed the methodological framework of the ACM-v1 logic model of asthma care, as described by Dima *et al*. ^23^, to map outcomes measured in each of the studies were mapped to nodes in the logic model. A mechanism of action could then be inferred, where not explicitly stated, by tracing a pathway through the model between study outcomes.

## Eligibility Criteria

The PICOS (Participant, Intervention, Comparison, Outcome, and Study Design) framework (24) was used to help develop eligibility criteria and search strategies ^24^ (Table 1) to identify primary-care based asthma CDSS implementation studies using data collected in a primary care setting. For identified studies, a peer-reviewed journal publication was not required: protocols, conference abstracts, clinical trial registrations, and funding reports were also included. Reports which were not available in English, or with no full-text accessible by the study team, were excluded. To build on the Matui *et al.* systematic review ^20^, we additionally restricted our search of peer-reviewed publications to those published in 2012 or later. Finally, we have excluded interventions for which the only studies solely assessed qualitative outcomes, as they did not allow any estimation of effect size or comparable impact to provide evidence of clinical improvement, necessary for further investment.

## Information Sources and Search Strategy

Five electronic databases (Embase via Ovid, PubMed, CENTRAL, the Health Technology Assessment (HTA) Database, and the ISRCTN registry of clinical trials) were searched to identify formal trial documents.

The core concepts used in the search strategies were a combination of asthma, CDSS interventions, and an interventional study design. We searched for any publications from 2012 to 2025, up until the search date of August 27^th^, 2025. The search terms are shown in Appendix A.

## Study Selection and Screening

Literature from ISRCTN registrations were separately retrieved and screened due to inefficient interoperability between the two platforms, and duplicates were manually removed.

All other retrieved studies were uploaded to the Covidence platform ^25^, where study selection and screening were conducted after automatic deduplication and exclusion. When multiple documents related to the same trial, a common identifier was recorded. Titles and abstracts were independently screened by two reviewers (HT and either BL or IS), followed by independent full-text screening. The concordance between reviewers was recorded at both stages. At the full text screening stage, the reasons for exclusion were also independently recorded and corroborated.

All uncertainties and disagreements during screening were resolved through discussions

## Data Extraction

We extracted data on decision system evidence-base, design, deployment, and evaluation, using Microsoft Excel. The data extraction template is presented in Appendix C. Data were extracted independently by HT and either BL or IS, with disagreements resolved through discussions with both reviewers. No quality appraisal was conducted as part of this review, as is not mandatory for scoping reviews ^26^, and as our primary aim was to identify the study designs tested rather than to critique their impact and effectiveness.

# Results

## Search Results

All sources were searched on August 27^th^, 2025. A PRISMA flow diagram was generated to show the exclusion of studies by stage and exclusion criteria (Appendix B). In total, 18 trials were included (Table 2). Six of the trials were based in the UK, five in the USA, five in Canada, and one in each of Spain, and Australia.

There were seven trials with only a single source of data. For three of these trials, the only document was a conference abstract. For other trials with a single document (n=4) this was a peer-reviewed feasibility trial results paper (n=3) or an RCT registration for an ongoing pilot trial (n=1).

For the other eleven trials with multiple documents, three had a protocol and nine had a results paper (two studies had both, and the final trial had neither – just two conference abstracts).

## Study Designs

Of the 18 trials, seven were randomised controlled trials (RCTs) and eleven were feasibility or pilot trials (Table 2).

In the feasibility/pilot trials, eight compared their intervention to usual care (including for the same patients before the intervention was delivered), one treated visits to the clinic after the introduction of the intervention as a continuous exposure, and one had no comparator (Table 2).

In the RCTs, the comparator was usual care in control practices or patients in five studies, and alternative versions of the intervention algorithm in the other two (including similar alerts on unrelated topics, or decision algorithms which did not incorporate certain key information).

For nine trials, the intervention was integrated into the EHR system, drawing data directly from patient records. For three trials, the system was a stand-alone software piece. Four trials used a web-based application. For one trial, the intervention was delivered through email. For one trial, we were not able to determine the platform of the system.

## Interventions

The intervention covered a range of objectives, and corresponding outputs, intended to support clinician decision making. These included risk prediction, treatment optimisation, and action plan delivery (Table 2). Ten trials referenced some element of prediction or classification, such as asthma control groups and asthma attack risk (STARRS-GM ^32^, GeoAsma ^47^, eAMS ^63^, PAAF ^28^, Lachman *et al.* ^38^, Millard *et al.* ^44^*,* ARRISA ^46^, a-GPS ^34^, ARRISA-UK ^37^, and MOXXI ADS ^31^). Twelve trials referenced highlighting recommended actions: treatment optimisation (n=7; The Breathing for Life Trial ^27^, PAAF ^28^, RAACENO ^29^, EPIPP ^30^, eAMS ^63^, MOXXI ADS ^31^, and STARRS-GM ^32^), and action plan generation (n=3; eAAP ^33^, AMOMS ^66^, and eAMS ^63^). Only one trial mentioned highlighting any of patient’s specific risk factors (a-GPS ^34^, which included flags for factors such as obesity and previous diagnosis of allergic rhinitis). Additionally, three trials mentioned providing graphical representations such as bar graphs and timelines (e-MEDRESP ^35^, Mukherjee *et al.* ^36^, and a-GPS ^34^), all of which were targeted at clinicians.

Five trials made use of previously developed interventions, and so did not describe their development in their own papers (PAAF ^28^, STARRS-GM ^32^, ARRISA-UK ^37^, Lachman *et al.* ^38^, and Lou *et al.* ^39^). For the remaining 14 trials, seven included some details pertaining to the process of their intervention development. The a-GPS ^34^ and eAAP ^33^ trials included clinical consultation, through surveys and focus group interviews. The e-MEDRESP trial also used clinical consultation, and a framework inspired by user-centred design, as described in a stand-alone publication ^40^. The eAMS trial’s CDSS ^63^ component was developed using evidence-based procedures and implementation guidance, and refined iteratively with stakeholders ^41,42^. The EPIPP intervention ^30^ was developed using 2012 Cochrane systematic review of audit and feedback by Ivers *et al*. ^43^. The intervention in the trial by Millard *et al.* ^44^ was developed using the US National Heart, Lung, and Blood Institute (NHLBI) Expert Panel Review (EPR) 3 for Asthma Diagnosis and Management ^45^. The ARRISA trial conducted data-driven algorithm refinement ^46^.

## Outcomes and Mechanisms of Action

Table 3 shows the measured outcomes in each trial, mapped to the Asthma Care Model (ACM-v1) logic model of asthma care, as described by Dima *et al*. ^23^. Two trials measured lung function or biomarkers, which were not specifically referenced in the ACM-v1 model, and have herein been categorised them under the node ‘asthma control’.

Fourteen trials measured changes in incidence or rates of severe asthma attacks, including use of systemic steroids, emergency department presentations, and inpatient admissions.

Six trials measured qualitative components relating to healthcare provider and patient satisfaction with the system (GeoAsma ^47^, a-GPS ^34^, EPIPP ^30^, ARRISA-UK ^37^, RAACENO ^29^, e-MEDRESP ^35^ and The Breathing for Life Trial ^27^). Six trials measured economic evaluations (GeoAsma ^47^, a-GPS ^34^, Lachman *et al.* ^38^, ARRISA-UK ^37^, RAACENO ^29^, and The Breathing for Life Trial ^27^), such as the Incremental cost-effectiveness ratio and incremental cost-utility ratio of the intervention, using Quality-adjusted Life Years (QALYs).

Most interventions were aimed at changing the behaviour of healthcare providers in order to impact patient outcomes (n=14), such as improving prescribing practices, delivering asthma action plans, and inviting patients to scheduled consultations. For four trials, however, the guidance of the system was the intervention, rather than the provision of the system, with healthcare providers using their own discretion about whether or not to follow the guidance (eAAP ^33^, Lou *et al.* ^39^, and The Breathing for Life Trial ^27^). Five trials also included endpoints reflecting patient behaviour change through adherence to treatment (GeoAsma ^47^, e-MEDRESP ^35^, PAAF ^28^, RAACENO ^29^, and ARRISA-UK ^37^). However, there was only evidence of the use of implementation or behaviour change theory in four studies. The eAMS study ^63^ used the Knowledge-to-Action Framework ^48^ and Theoretical Domains Framework ^49^. The e-MEDRESP study ^35^ used a user-centred design framework ^50^. The ARRISA-UK trial ^37^ used the Process Evaluation of Complex Interventions framework ^51^. Finally, the PAAF trial used both the Diffusion of Innovation theory ^52^ and Theoretical Domains Framework ^49^, however they were only used retrospectively to explore why the system uptake was poor, rather than proactively to prevent it ^28^.

## Study Results

Three out of ten feasibility or pilot trials with some reported results looked at system use - eAMS ^64^, PAAF ^28^, and e-MEDRESP ^35^. In the PAAF trial, the system was only used for 12/143 (8%) of patients who were eligible ^28^. The eAMS ^64^ and e-MEDRESP ^35^ trials reported that the system was used in 20% and 34% of opportunities, respectively (Table 4), and there was positive feedback from users in qualitative evaluations. The e-MEDRESP trial also looked engagement over time, and found a rapid decline in use throughout the trial ^35^. All feasibility or pilot trials excluding eAMS ^64^ assessed at least one patient outcome, and of these ten, eight reported some positive impact.

In the seven RCTs with some results reported (a conference abstract and/or results paper), three reported outcomes related to the feasibility of the intervention, and three reported outcomes related to uptake and acceptability (Table 5). The RAACENO trial reported that the user did not follow the recommendation to step-up treatment in approximate 25% of cases in either the intervention or control algorithm arm ^53^, and the MOXXI ADS trial found their out-of-control alerts did not prompt the user to open the system in 60% of cases ^31^.

Three trials reported asthma action plan delivery relative to a comparator arm (STARRS-GM ^32^, AMOMS ^66^, and PAAF ^28^), and all observed an increase. Furthermore, of the five trials targeting any aspect of health care professional behavioural care (Table 3), three (STARRS-GM ^32^, AMOMS ^66^, and PAAF ^28^) saw impact on patient care.

Similarly, both trials reporting on prescribed inhaled corticosteroids (ICS) to short-acting beta-2 agonists (reliever inhalers; SABA) ratio (Lachman *et al.* ^38^ and MOXXI ADS ^31^) achieved positive results in these outcomes. In general, five of the nine trials targeting prescribed drugs (Table 3) saw impact (STARRS-GM ^32^, PAAF ^28^, Lachman *et al.* ^38^*,* Millard *et al.* ^44^, and EPIPP ^30^).

Only two trials (AMOMS ^66^ and PAAF ^28^) in four (the others being RAACENO ^54^ and a-GPS ^34^) which aimed to improve asthma control were successful, and there as no improvement in the trial which looked at asthma quality of life (RAACENO ^54^).

Results for trials relating to asthma attack incidence were more varied depending on definition. Seven trials looked to decrease oral steroid use, of which three were successful (Mukherjee *et al.* ^36^, PAAF ^28^, and eAAP – but only in children ^33^), three observed no change (e-MEDRESP ^35^, RAACENO ^54^, and ARRISA-UK ^55^), and one observed an increase (ARRISA ^46^), but with emergency presentations decreasing. This raises crucial questions about the specific changes to healthcare provision resulting from the intervention, and if we can ascertain whether patients are being treated earlier and thus not needing emergency care, or if some other mechanism explains these results.

Furthermore, grouping trials instead by intervention type (Table 2), we observed that neither of the studies focussed on treatment optimisation observed any positive impact on patient care (The Breathing for Life Trial ^27^ and RAACENO ^54^*.)*.The trials primarily using alerts had very mixed results, with positive changes to prescribing (ARRISA ^46^, ARRISA-UK ^55^, MOXXI ADS ^31^, Lou *et al.* ^39^, and EPIPP ^30^). In contrast, all of the studies focussed on clinical risk prediction observed improvements (PAAF ^28^, Lachman *et al.* ^38^*,* and Millard *et al.* ^44^*).*

# Discussion

## Summary of Results

This scoping review identified 18 asthma CDSS implementation trials conducted over the past decade, spanning feasibility studies, pilot evaluations, and randomised controlled trials across six countries. Despite substantial diversity in intervention purpose, technical design, and integration with clinical workflows, consistent patterns emerged. Most systems aimed to influence clinician behaviour through risk prediction, treatment optimisation, or action-plan delivery, yet engagement with the systems was generally low and often declined rapidly over time. Although some trials demonstrated improvements in process measures, such as action-plan delivery or ICS–SABA ratios, few produced meaningful improvements in asthma control, quality of life, or exacerbation rates. The scarcity of behaviour-change or implementation theory underpinning intervention design likely contributed to these limited effects.

## Results in Context

In the full review stage, there were a high number of conflicts (12%) between reviewers. The primary source of conflict was in deciding whether an intervention was eligible based on how (or whether) individual patient data was leveraged. For example, a paper was excluded which issued reminders to check whether a patient was eligible for an influenza vaccination to all patients over a certain age, but did not utilise any historic data to confirm eligibility. Similarly, it was not always straight-forward to classify whether data was collected in the strictly primary care setting, such as the eAMS study ^63^, which utilised data collected from patients in the waiting room prior to the primary care appointment (this study was included).

Furthermore, the review by Matui *et al.* ^20^ used the definition by Wyatt *et al.* requiring ‘two or more items of patient data to generate case-specific advice’ ^56^, however this was very difficult to determine for many studies, and some degree of subjectivity was required. We decided to be as inclusive as possible in our inclusion criteria, and include trials for which there was insufficient evidence to include or exclude.

There was also extensive discussion during this stage about what constituted ‘advice’, and whether decision support specifically indicated the generation of new data (such as predictions, classifications, or recommendations) or whether presentation and summary of data towards efficient decision making would also justify this definition. We also included trials which were did not issue guidance at the point of care, such as in the a-GPS system ^34^, in contrast to reviews in other conditions ^57^.

In the review by Matui *et al.* ^20^, most studies reported poor engagement with the intervention, For example, one study reported that only 7% of the decision support comments were read ^58^. Uptake of the system continued to be a barrier in our review, as even the MOXXI ADS alert intervention, which managed to achieve significant improvement in ICS/SABA ratio compared to the control arm, was not opened in 60% of cases in which it was issued ^31^. Only two of the eight studies in the Matui review reported exacerbation rates, and only one of those managed to achieve a significant reduction in asthma exacerbations (but not in use of oral steroids) ^9^. In our review, asthma attacks were a more commonly used outcome, with 16 of 21 trials including this as an outcome. However, most trials failed to achieve a significant reduction in the intervention arm. These findings are in line with those reported beyond asthma – very few CDSS intervention trials were able to achieve any substantial improvements in the processes of care targeted in the intervention ^57^.

## Implications for Future Research

There is need to strengthen the design and evaluation of CDSS interventions. Firstly, intervention development should be informed by theory, such as behaviour change frameworks like the COM-B ^59^, Theoretical Domains Framework ^49^, and Knowledge to Action ^48^. Embedding a priori mechanisms of action would allow clearer hypotheses, targeted outcomes, and stronger logic models for evaluation. Reporting transparency also needs to be improved, with several studies providing insufficient information for clarity on the intervention development, delivery mode and data sources.

Poor system uptake and rapid declines in usage throughout trials were recurring issues. As noted in Table 2, the vast majority of the interventions in this review were passive tools, which required the user to actively alter their workflow in order to even receive the information that might inform a change in care. The studies which included process evaluation components highlighted how infrequently this occurred. Mixed-methods process evaluation is an essential element of trial design in order to understand the determinants of CDSS adoption and sustainability, but clinical input at the design stage may promote a more seamless integration with current workflows. Guidance such as the UK MRC clinical intervention development methodology ^60^, and Six steps in quality intervention development (6SQuID) framework ^61^ can guide developers through theory-informed best practices.

This study focussed on CDSS using data that is already collected routinely in primary care. However, as remote patient monitoring devices, such as smart peak flow measures, become more feasible for use in routine care, further work is needed to investigate their cost-effectiveness for improving decision support.

Finally, integration with primary care workflows is crucial for successful intervention implementation. Embedding CDSS into EHR systems is preferable, but may require collaboration with policymakers and software vendors to implement.

## Strengths and Limitations

One of the strengths of this study was that we conducted comprehensive searches by using multi-platform search strategies and including trials which did not have peer-reviewed journal articles (for protocols or results). We identified all conference abstracts or grey literature such as clinical trials registrations, which enabled to mitigate the risk of publication bias and review the state-of-the-art approaches being used in ongoing trials. However, the reporting herein for five of the 18 trials relied solely on such grey literature. This restricted the depth of extraction, and as such some classification decisions required interpretation from the reviewers, which inevitably introduced some subjectivity.

We also used a broad, multi-platform search strategy, which identified a wide range of documents. Furthermore, fewer than 20% of the documents were duplicates across platforms, despite strongly aligned search criteria, which highlighted the strength of our database selection.

Our study was novel in its mapping of the study outcomes to the ACM-v1 logic model by Dima *et al*. ^23^. This provided conceptual clarity and allowed us to infer mechanisms of action even when studies did not articulate them themselves. This was particularly valuable as most trials offered minimal explanation of behavioural or implementation logic, or reference to any theory underpinning the development of their interventions.

The study also included c**lear identification of barriers to real-world CDSS impact, highlighting** cross-study themes such as low uptake, limited behaviour change, and weak integration with workflows.

This study did not include a quality assessment of the included studies, as the focus of the study was on the variety of methods included in the trials, including the range of interventions and outcomes, rather than to provide evidence for the effectiveness of specific interventions.

The heterogeneity of the trials, however, meant that evidence synthesis was very difficult, and we were not able to describe any associations between facets of the intervention and the outcomes.

## Conclusion

Across 18 identified recent asthma CDSS implementation trials, very few were able to demonstrate meaningful improvements in patient outcomes. Successful implementation requires more than accurate algorithms: systems must be seamlessly embedded in workflows, transparently convey their purpose and limitations, and support sustainable clinical behaviour change. Future research should prioritise rigorous development processes, theory-driven implementation strategies, and long-term evaluation of real-world use. Without these elements, CDSSs are unlikely to deliver the clinical benefit necessary for widespread adoption in asthma care.

# References

1. Asthma UK. *UK Asthma Death Rates among Worst in Europe*. (2017).

2. Iacobucci, G. Asthma deaths rise 33% in past decade in England and Wales. *British Medical Journal* **366**, l5108 (2019).

3. Sutton, R. T. *et al.* An overview of clinical decision support systems: benefits, risks, and strategies for success. *npj Digital Medicine* **3**, 17 (2020).

4. Poels, P. J. *et al.* Spirometry expert support in family practice: a cluster-randomised trial. *Prim Care Respir J* **18**, 189–197 (2009).

5. Smith, J. R. *et al.* The at-risk registers in severe asthma (ARRISA) study: A cluster-randomised controlled trial examining effectiveness and costs in primary care. *Thorax* **67**, 1052–1060 (2012).

6. Fiks, A. G. *et al.* Impact of Electronic Health Record-Based Alerts on Influenza Vaccination for Children With Asthma. *Pediatrics* **124**, 159–169 (2009).

7. Kattan, M. *et al.* A Randomized Clinical Trial of Clinician Feedback to Improve Quality of Care for Inner-city Children With Asthma. *Pediatrics* **117**, e1095–e1095 (2006).

8. Eccles, M. *et al.* Effect of computerised evidence based guidelines on management of asthma and angina in adults in primary care: cluster randomised controlled trial. *BMJ* **325**, 941 (2002).

9. McCowan, R. G. N., I. W. Ricketts, F. C. Warner, G. Hoskins, G. E. Thomas, C. Lessons from a randomized controlled trial designed to evaluate computer decision support software to improve the management of asthma. *Medical Informatics and the Internet in Medicine* **26**, 191–201 (2001).

10. Tierney, W. M. *et al.* Can Computer-Generated Evidence-Based Care Suggestions Enhance Evidence-Based Management of Asthma and Chronic Obstructive Pulmonary Disease? A Randomized, Controlled Trial. *Health Services Research* **40**, 477–498 (2005).

11. Global Initiative for Asthma. *Global Strategy for Asthma Management and Prevention (2020)*. *Global Strategy for Asthma Management and Prevention* (2020).

12. British Thoracic Society & SIGN. *British Guideline on the Management of Asthma (2019 Edition)*. (2019).

13. National Institute of Health and Care Excellence. *Asthma: Diagnosis, Monitoring and Chronic Asthma Management NICE Guideline*. (2020).

14. Dekker, F. W., Ramspek, C. L. & Van Diepen, M. Most clinical risk scores are useless. *Nephrology Dialysis Transplantation* vol. 32 752–755 Preprint at https://doi.org/10.1093/ndt/gfx073 (2017).

15. Roshanov, P. S. *et al.* Features of effective computerised clinical decision support systems: meta-regression of 162 randomised trials. *BMJ (Clinical research ed.)* **346**, f657 (2013).

16. Cai, C. J., Winter, S., Steiner, D., Wilcox, L. & Terry, M. ‘Hello AI’: Uncovering the Onboarding Needs of Medical Practitioners for Human–AI Collaborative Decision-Making. in *Proceedings of the ACM on Human-computer Interaction* 104 (2019). doi:10.1145/3359206.

17. Carroll, C. *et al.* Involving users in the design and usability evaluation of a clinical decision support system. *Computer Methods and Programs in Biomedicine* **69**, 123–135 (2002).

18. Hocking, J. S. *et al.* The impact of removing financial incentives and/or audit and feedback on chlamydia testing in general practice: A cluster randomised controlled trial (ACCEPt-able). *PLOS Medicine* **19**, e1003858 (2022).

19. Rousseau, N., McColl, E., Newton, J., Grimshaw, J. & Eccles, M. Practice based, longitudinal, qualitative interview study of computerised evidence based guidelines in primary care. *BMJ* **326**, 314 (2003).

20. Matui, P., Wyatt, J. C., Pinnock, H., Sheikh, A. & McLean, S. Computer decision support systems for asthma: a systematic review. *npj Prim Care Resp Med* **24**, 1–10 (2014).

21. Tibble, H. *Scoping Review Protocol for Primary Care Clinical Decision Support Trials for Asthma*. https://osf.io/ptck6/ (2024).

22. Tricco, A. C. *et al.* PRISMA Extension for Scoping Reviews (PRISMA-ScR): Checklist and Explanation. *Ann Intern Med* **169**, 467–473 (2018).

23. Dima, A. L., de Bruin, M. & Van Ganse, E. Mapping the Asthma Care Process: Implications for Research and Practice. *The Journal of Allergy and Clinical Immunology: In Practice* **4**, 868–876 (2016).

24. Richardson, W. S., Wilson, M. C., Nishikawa, J. & Hayward, R. S. The well-built clinical question: a key to evidence-based decisions. *ACP J Club* **123**, A12-13 (1995).

25. Covidence systematic review software. Veritas Health Innovation.

26. Pollock, D. *et al.* “How-to”: scoping review? *Journal of Clinical Epidemiology* **176**, 111572 (2024).

27. Murphy, V. E. *et al.* Effect of asthma management with exhaled nitric oxide versus usual care on perinatal outcomes. in *European Respiratory Journal* vol. 60 (European Respiratory Society, 2022).

28. McFarlane, M. L., Morra, A., Podgers, D., Barber, D. & Lougheed, M. D. Impact of a Novel Electronic Medical Record–Integrated Electronic Form (Provider Asthma Assessment Form) and Severe Asthma Algorithm in Primary Care: Single-Center, Pre- and Postobservational Study. *JMIR Form Res* **9**, e74043–e74043 (2025).

29. Turner, S. *et al.* Reducing Asthma Attacks in Children using Exhaled Nitric Oxide as a biomarker to inform treatment strategy: a randomised trial (RAACENO). *Trials* **20**, 573 (2019).

30. MacBride-Stewart, S., Marwick, C., Ryan, M. & Guthrie, B. Feedback of actionable individual patient prescription data to improve asthma prescribing: pragmatic cluster randomised trial in 233 UK general practices. *Br J Gen Pract* **72**, e627–e633 (2022).

31. Tamblyn, R. *et al.* Evaluating the impact of an integrated computer-based decision support with person-centered analytics for the management of asthma in primary care: a randomized controlled trial. *Journal of the American Medical Informatics Association* **22**, 773–783 (2015).

32. Chakrabarti, B. *et al.* The feasibility and impact of implementing a computer-guided consultation to target health inequality in Asthma. *npj Prim. Care Respir. Med.* **33**, 1–7 (2023).

33. Kuhn, L., Tapp, H., Reeves, K. & Dulin, M. The Impact of an Integrated Electronic Medical Record Asthma Action Plan Decision Support Tool. in *B27. VIRAL INFECTION AND PEDIATRIC ASTHMA* A2637–A2637 (American Thoracic Society, 2015). doi:10.1164/ajrccm-conference.2015.191.1_MeetingAbstracts.A2637.

34. Seol, H. Y. *et al.* Artificial intelligence-assisted clinical decision support for childhood asthma management: A randomized clinical trial. *PLOS ONE* **16**, e0255261 (2021).

35. Yousif, A., Lemière, C., Forget, A., Beauchesne, M.-F. & Blais, L. Feasibility of implementing a web-based tool built from pharmacy claims data (e-MEDRESP) to monitor adherence to respiratory medications in primary care. *Current Medical Research and Opinion* **38**, 2055–2067 (2022).

36. Mukherjee, M. *et al.* Deploying an asthma dashboard to support quality improvement across a nationally representative sentinel network of 7.6 million people in England. *npj Prim. Care Respir. Med.* **34**, 18 (2024).

37. Smith, J. R. *et al.* At-risk registers integrated into primary care to stop asthma crises in the UK (ARRISA-UK): Study protocol for a pragmatic, cluster randomised trial with nested health economic and process evaluations. *Trials* **19**, 466–466 (2018).

38. Lachman, B. S. & Pengetnze, Y. Improved Asthma Outcomes from Use of Predictive Modeling As Part of a System of Care. in *Journal of Allergy and Clinical Immunology* vol. 139 AB191 (Elsevier, 2017).

39. Lou, Y. *et al.* The impact of care management for high-risk pediatric asthmatics on healthcare utilization. *Journal of Asthma* **58**, 133–140 (2021).

40. Yousif, A. *et al.* Development of a Web-Based Tool Built From Pharmacy Claims Data to Assess Adherence to Respiratory Medications in Primary Care. *Respiratory Care* **65**, 1355–1366 (2020).

41. Kouri, A., Boulet, L.-P., Kaplan, A. & Gupta, S. An evidence-based, point-of-care tool to guide completion of asthma action plans in practice. *Eur Respir J* **49**, 1602238 (2017).

42. Gupta, S., Wan, F. T., Hall, S. E. & Straus, S. E. An asthma action plan created by physician, educator and patient online collaboration with usability and visual design optimization. *Respiration* **84**, 406–415 (2012).

43. Ivers, N. *et al.* Audit and feedback: effects on professional practice and healthcare outcomes - Ivers, N - 2012 | Cochrane Library. https://www.cochranelibrary.com/cdsr/doi/10.1002/14651858.CD000259.pub3/full.

44. Millard, M. *et al.* Impact of an EMR Documentation Template as Clinical Decision Support for Outpatient Asthma Management. *CHEST* **144**, 68A (2013).

45. National Heart, Lung, and Blood Institute. *Guidelines for the Diagnosis and Management of Asthma 2007 (EPR-3)*. https://www.nhlbi.nih.gov/health-topics/guidelines-for-diagnosis-management-of-asthma (2007).

46. Smith, J. R. *et al.* The at-risk registers in severe asthma (ARRISA) study: A cluster-randomised controlled trial examining effectiveness and costs in primary care. *Thorax* **67**, 1052–1060 (2012).

47. Conde, A. M. *GeoAsma: Pilot and Evaluation of a Clinical Decision Support System and a Mobile App for the Management of Asthma Patients.* https://clinicaltrials.gov/study/NCT05639101 (2022).

48. Graham, I. D. *et al.* Lost in knowledge translation: Time for a map? *Journal of Continuing Education in the Health Professions* **26**, 13–24 (2006).

49. Michie, S. Making psychological theory useful for implementing evidence based practice: a consensus approach. *Quality and Safety in Health Care* **14**, 26–33 (2005).

50. Garrett, J. J. *The Elements of User Experience: User-Centered Design for the Web*. (New Riders [u.a.], New York, 2008).

51. Moore, G. F. *et al.* Process evaluation of complex interventions: Medical Research Council guidance. *BMJ* **350**, h1258 (2015).

52. Dearing, J. W. & Cox, J. G. Diffusion Of Innovations Theory, Principles, And Practice. *Health Affairs* **37**, 183–190 (2018).

53. Lawrie, L., Turner, S., Cotton, S. C., Wood, J. & Morgan, H. M. A qualitative process evaluation within a clinical trial that used healthcare technologies for children with asthma–insights and implications. *PLOS ONE* **18**, e0280086 (2023).

54. Turner, S. *et al.* Reducing asthma attacks in children using exhaled nitric oxide (RAACENO) as a biomarker to inform treatment strategy: a multicentre, parallel, randomised, controlled, phase 3 trial. *The Lancet Respiratory Medicine* **10**, 584–592 (2022).

55. Wilson, A. M. *et al.* At-risk registers integrated into primary care to stop asthma crises in the UK (ARRISA-UK). in *Thorax* vol. 79 A76–A76 (BMJ Publishing Group Ltd, 2024).

56. Wyatt, J. & Spiegelhalter, D. Field trials of medical decision-aids: potential problems and solutions. *Proc Annu Symp Comput Appl Med Care* 3–7 (1991).

57. Kwan, J. L. *et al.* Computerised clinical decision support systems and absolute improvements in care: meta-analysis of controlled clinical trials. *BMJ* **370**, m3216 (2020).

58. Kuilboer, M. M. *et al.* Computed Critiquing Integrated into Daily Clinical Practice Affects Physicians’ Behavior. *Methods of Information in Medicine* **45**, 447–454 (2018).

59. Michie, S., van Stralen, M. M. & West, R. The behaviour change wheel: A new method for characterising and designing behaviour change interventions. *Implementation Science* **6**, 42 (2011).

60. Skivington, K. *et al.* A new framework for developing and evaluating complex interventions: update of Medical Research Council guidance. *BMJ* **374**, n2061 (2021).

61. Wight, D., Wimbush, E., Jepson, R. & Doi, L. Six steps in quality intervention development (6SQuID). *J Epidemiol Community Health* **70**, 520–525 (2016).

62. Wyatt, J. & Spiegelhalter, D. Field trials of medical decision-aids: potential problems and solutions. *Proc Annu Symp Comput Appl Med Care* 3–7 (1991).

63. Gupta, S. *et al.* The Electronic Asthma Management System (eAMS) improves primary care asthma management. *Eur Respir J* **53**, 1802241 (2019).

64. Lam Shin Cheung, J. *et al.* A system uptake analysis and GUIDES checklist evaluation of the Electronic Asthma Management System: A point-of-care computerized clinical decision support system. *Journal of the American Medical Informatics Association* **27**, 726–737 (2020).

65. *The Electronic Asthma Action Plan System for Implementation in Primary Care (eAAPS)*. https://clinicaltrials.gov/study/NCT01070095.

66. Taite, A. *et al.* Enabling asthma management and outcomes monitoring through standardized EMR data and eTools. in *European Respiratory Journal* vol. 50 (European Respiratory Society, 2017).

67. Taite, A. *et al.* Use of an electronic asthma management and outcomes monitoring system (AMOMS) and integrated etools for patient care and quality improvement. in *Canadian Journal of Respiratory, Critical Care, and Sleep Medicine* vol. 2 90–121 (Taylor & Francis, 2018).

68. M L McFarlane, Morra, A., Podgers, D., Barber, D. & Lougheed, M. D. Promoting Evidence-based Asthma Care Using Digital Knowledge Translation Tools-Impact of the Provider Asthma Assessment Form. in *D22. ADVANCES IN HEALTH SERVICES RESEARCH IN ASTHMA* A7023–A7023 (2024).

69. Murphy, V. E. *et al.* The Breathing for Life Trial: a randomised controlled trial of fractional exhaled nitric oxide (FENO)-based management of asthma during pregnancy and its impact on perinatal outcomes and infant and childhood respiratory health. *BMC Pregnancy Childbirth* **16**, 111 (2016).

70. Smith, J. R. *et al.* The At-Risk Registers in Severe Asthma (ARRISA) Study: a cluster-randomised controlled trial in primary care. in *Thorax* vol. 65 A62–A62 (BMJ Publishing Group Ltd, 2010).

71. Turner, S. *et al.* Reducing Asthma Attacks in Children using Exhaled Nitric Oxide as a biomarker to inform treatment strategy: a randomised trial (RAACENO). in *Idiopathic interstitial pneumonias* RCT2899 (European Respiratory Society, 2021). doi:10.1183/13993003.congress-2021.RCT2899.

72. Juhn, Y. *et al.* Asthma-Guidance and Prediction System (a-GPS) As a Precision Asthma Care Tool. in *Programs and Abstracts of Papers to be Presented During Scientific Sessions: 2020 AAAAI Annual Meeting* vol. 145 AB210 (2020).

73. *Developing and Implementing Asthma-Guidance and Prediction System (a-GPS) for Better Asthma Management*.

74. Smith, J. R. *et al.* Initial process evaluation findings from the at-risk registers integrated into primary care to stop asthma crises in the UK (ARRISA-UK) trial: practice characteristics, engagement and early experiences of the intervention. in *Thorax* vol. 74 A184–A185 (BMJ Publishing Group Ltd, 2019).

75. *Computer-Based Decision Support in Managing Asthma in Primary Care*. https://clinicaltrials.gov/study/NCT00170248.

76. *Electronic Prescription Data to Improve Primary Care Prescribing (EPIPP)*. https://clinicaltrials.gov/study/NCT02512198.

# Tables

Table 1: PICOS Framework Eligibility Criteria

|  | Inclusion Criteria |
| --- | --- |
| *Participants* | Individuals formally diagnosed with asthma, or the healthcare professionals attending to them in the primary care setting. |
| *Intervention* | CDSS for use in primary care and community asthma management, which made use of individual patient data. CDSSs can be either powered by data-driven algorithms or expert guidance, but must be delivered using individual patient data ^62^. Herein, we specified that they must be used by a healthcare professional, using a digital system (computer application, mobile device application, or web application).  CDSS solely for the task of diagnosing asthma were excluded in this study, as were interventions which were focussed on the collection of new data from all participants, without any data-driven selection criteria or filtering.  Data for the intervention was not collected in the home setting or the secondary care setting. |
| *Comparison* | There were no requirements regarding the comparison. For controlled studies, for example, the comparison could be standard of care or an alternative intervention. For feasibility studies, no comparison arm at all was required. |
| *Outcome* | Any quantitative outcomes were permitted, including those relating to system acceptability, patient outcomes, medical care and service provision, and health economics. Qualitative outcomes were also recorded, so long as at least one quantitative outcome was reported. |
| *Study Design* | Clinical trials, including both randomised controlled trials and single-arm feasibility studies.  The intervention must be either accessed in a natural setting (regular time-points or patient-based triggers, rather than specified study timepoints) or be optional to apply (e.g. there may be a distinction between the per protocol and intent to treat populations discussed). |

Table 2: Characteristics of Included Trials

| **Trial Type** | **Project Name (Clinical Trial Registration) [Country]** | **Protocol Published Year (Citation)** | **Results Published Year**  **(Citation)** | **Other Citations** | **Intervention** | **Workflow Integration** | **Comparator** |
| --- | --- | --- | --- | --- | --- | --- | --- |
| Feasibility or Pilot Trial | STARRS-GM  (N/A)  [UK] |  | 2023 ^32^ |  | **Risk classification and treatment optimisation:** Response-mediated structured consultation guide, based on treatment and asthma control, and searchable register of high-risk patients. | Stand-alone software, with manual access at point of care or from manual query. | Usual care – before intervention |
|  | GeoAsma  (NCT05639101)  [Spain] |  |  | clinicaltrials.gov registration for study commencing in 2020 ^47^ | **Risk classification**:  Clinical risk prediction web system and self-management mobile application for patients, based on electronic health record data. | Web-based application, with manual access at point of care. | Usual care – before intervention |
|  | eAMS  (NCT01070095)  [Canada] |  | 2019 ^63^,  2020 ^64^ | clinicaltrials.gov registration for study commencing in 2012 ^65^ | **Risk classification, treatment optimisation and action-plan generation**: Treatment optimisation algorithm, based on treatment and patient reported data collected in waiting room, and action plan generation software. | Integrated into the EHR system, with manual access at point of care. | None |
|  | AMOMS  (N/A)  [Canada] |  |  | Conference abstracts from 2017 ^66^ and 2018 ^67^ | **Action-plan generation**:  EHR-integrated software for action plan generation, resource signposting, and assessment system | Integrated into the EHR system, with manual access at point of care. | None, but number of visits treated as an exposure to intervention. |
|  | Mukherjee *et al. **  (N/A)  [UK] |  | 2024 ^36^ |  | **Reporting**: Web-based dashboard of practice level data, extracted from electronic health records. | Web-based application, with manual access at any time. | Usual care |
|  | e-MEDRESP  (N/A)  [Canada] |  | 2022 ^35^ | Intervention development paper ^40^ | **Reporting**: Web-based adherence dashboard, based on prescription refills. | Web-based application, with manual access at point of care. | Usual care – before intervention |
|  | eAAP  (N/A)  [USA] |  |  | Conference abstract from 2015 ^33^ | **Action-plan generation**:  Action plan generation software (data inputs not specified). | Integrated into the EHR system, with manual access at the point of care. | Usual care – before intervention |
|  | PAAF  (N/A)  [Canada] |  | 2025 ^28^ | Conference abstract from 2024 ^68^ | **Risk classification**:  Clinical risk prediction system, based on diagnosis, family history, smoking history, asthma severity, occupational history, respiratory medications, asthma control, care, management and referrals, asthma action plan, asthma control zone, pulmonary function tests, and assessment tools. | Integrated into the EHR system, with manual access at the point of care. | Usual care – before intervention |
|  | Lachman *et al. **  (N/A)  [USA] |  |  | Conference abstract from 2017 ^38^ | **Risk classification**:  Risk prediction model and alerts (data inputs unclear) | Integrated into the EHR system, for use at point of care (unclear if manually or automatically triggered) | Usual care – before intervention |
|  | Millard *et al. **  (N/A)  [USA] |  |  | Conference abstract from 2013 ^44^ | **Risk classification**:  Data extraction algorithm for determining asthma control from electronic health records. | Integrated into the EHR system (access unclear) | Usual care |
|  | Lou *et al. **  (N/A)  [USA] |  | 2021 ^39^ |  | **Alerts**: Alerts for patient recall, vaccinations, and post-discharge follow-up, based on electronic health records. | Unclear | Usual care – before intervention |
| Randomised Controlled Trial | The Breathing for Life Trial  (ACTRN12613000202763)  [Australia] | 2016 ^69^ | 2022 ^27^ |  | **Treatment optimisation**: Treatment optimisation algorithm, based on ACQ and FENO measurements. | Stand-alone software, accessed at point of care. | Usual care |
|  | ARRISA  (ISRCTN36918958)  [UK] |  | 2012 ^46^ | Conference abstract from 2010 ^70^ | **Risk classification and alerts**: Alerts flagging at-risk patients, based on prescriptions, A&E presentations, hospital admissions, and psychosocial problems noted by manual review. | Active alerts, from software integrated into the EHR system, triggered at point of care. | Usual care |
|  | RAACENO  (ISRCTN67875351)  [UK] | 2019 ^29^ | 2022 ^54^, 2023 ^53^ | Conference abstract from 2021 ^71^ | **Treatment optimisation**: Treatment optimisation algorithm, based on ACT/CACT, FENO, current treatment, and adherence. | Web-based application, accessed at at quarterly study follow-up appointments. | (Alternative) treatment optimisation algorithm |
|  | a-GPS  (NCT02865967)  [USA] |  | 2021 ^34^ | Conference abstract from 2020 ^72^, and clinicaltrials.gov registration for study commencing in 2016 ^73^ | **Risk classification and reporting**: Quarterly reports on care quality and risk of attacks for each asthma patient, based on ACT, spirometry, unscheduled asthma visits in primary and secondary care, and more. | Stand-alone software, with updated reports issued every three months. | Usual care |
|  | ARRISA-UK  (ISRCTN95472706)  [UK] | 2018 ^37^ |  | Conference abstracts from 2019 ^74^ and 2024 ^55^ | **Risk classification and alerts**: Alerts flagging at-risk patients, based on prescriptions, A&E presentations, hospital admissions, and psychosocial problems noted by manual review. | Active alerts, from software integrated into the EHR system, triggered at point of care. | Usual care |
|  | MOXXI ADS  (NCT00170248)  [Canada] |  | 2015 ^31^ | clinicaltrials.gov registration for study commencing in 2006 ^75^ | **Risk classification, alerts and treatment optimisation**: Alerts flagging at-risk patients, with personalized management recommendations, based on treatment, comorbidities, and unscheduled asthma care. | Active alerts, from software integrated into the EHR system, triggered at point of care. | Usual care |
|  | EPIPP  (NCT02512198)  [UK] |  | 2022 ^30^ | clinicaltrials.gov registration for study commencing in 2015 ^76^ | **Alerts and treatment optimisation**: Alerts for potentially inappropriate prescribing of bronchodilator inhalers | Emails (three times in a 13-month period) | Alerts on an unrelated prescribing subject |

* No project name – lead author of sole publication listed

ACQ = Asthma Control Questionnaire, SABA = Short-Acting Beta-2 Agonist, A&E = Accident and Emergency Department, ACT = Asthma Control Test, CACT = Childhood Asthma Control Test, FENO = Fractional Exhaled Nitric Oxide.

Table 3: Trial Outcomes Examined

| **Project Name** | **Logic model of asthma care (ACM-v1) node** | | | | | |
| --- | --- | --- | --- | --- | --- | --- |
|  | **Prescribed Drug Exposure (9 trials)** | **Asthma Control (9 trials)** | **Severe asthma exacerbation (16 trials)** | **Quality of Life (5 trials)** | **Health care professional behavioural care (5 trials)** | **Adherence – regular and correct inhaler use (9 trials)** |
| **STARRS-GM** ^32^ | - ICS Prescriptions - Spacers given |  |  |  | - Personal action plans given - Adherence discussed - Invited to smoking cessation services |  |
| **GeoAsma** ^47^ |  | - Asthma control - Respiratory function - Immune response | - Severe exacerbations | - Health-related quality of life - Quality adjusted life years |  | - Treatment adherence |
| **eAMS** ^63^ |  |  |  |  | - Asthma action plan delivery |  |
| **AMOMS** ^66^ |  | - Asthma control |  |  | - Asthma action plan delivery |  |
| **Mukherjee *et al. **** ^36^ |  |  | - Severe exacerbations - Use of systemic steroids - Asthma related emergency department visits - Asthma related hospitalisations |  |  |  |
| **e-MEDRESP** ^35^ |  |  | - Use of oral steroids |  |  | - Treatment adherence - SABA usage |
| **eAAP** ^33^ |  |  | - Severe exacerbations - Use of systemic steroids - Asthma related emergency department visits - Asthma related hospitalisations |  |  |  |
| **PAAF** ^28^ | - Combined ICS and reliever inhaler use | - Asthma control | - Use of systemic steroids - Asthma related emergency department visits - Asthma related hospitalisations |  | - Asthma action plan delivery - Pulmonary function monitoring - Inhaler technique assessments - Invited to smoking cessation services - Referral to severe asthma services | - Treatment adherence |
| **Lachman *et al.*** *** ^38^ | - ICS Prescriptions |  | - Asthma related emergency department visits - Asthma related hospitalisations |  |  | - ICS/SABA ratio |
| **Millard *et al.*** *** ^44^ | - Regimen changes |  | - Out-of-control asthma events |  |  |  |
| **Lou *et al.*** *** ^39^ |  |  | - Urgent Care - Hospitalisation |  |  |  |
| **The Breathing for Life Trial** ^27^ |  |  | - Severe exacerbations |  |  |  |
| **ARRISA** ^46^ |  |  | - Severe exacerbations - Asthma related hospitalisations - Use of systemic steroids |  |  |  |
| **RAACENO** ^54^ | - ICS Dose Prescribed | - Asthma control - Respiratory function - Biomarkers | - Use of oral steroids for asthma attacks - Unscheduled healthcare assessment | - Paediatric Asthma Quality of Life |  | - Treatment adherence |
| **a-GPS** ^34^ |  | - Asthma control | - Asthma related emergency department visits |  |  |  |
| **ARRISA-UK** ^37^ | - SABA prescriptions issued - Regimen changes | - Asthma control | - Severe exacerbations - Use of systemic steroids |  | - Asthma action plan delivery - Peak flow diary delivery - Inhaler technique assessments - Invited to smoking cessation services - Flu vaccinations | - adherence to medication |
| **MOXXI ADS** ^31^ |  |  | - Out-of-control episodes |  |  | - ICS/SABA ratio |
| **EPIPP** ^30^ | - LABAs with subtherapeutic or no ICS |  |  |  |  | - Excess SABA use |

* No project name – lead author of sole publication listed

Table 4: Feasibility and Pilot Trial Results

| **Comparator** | **Project Name** | **Feasibility** | **Uptake and Acceptability** | **Impact** |
| --- | --- | --- | --- | --- |
| None | eAMS ^63^ |  | Clinicians opened the CCDSS in 19.8% possible instances in which CCDSS recommendations were available. Clinician user feedback was generally positive. Action plans were created in 59.0% of CCDSS openings. |  |
| Exposure to Intervention | AMOMS ^66^ |  |  | The percentage of patients with a) controlled asthma, and b) an asthma action plan increased with the number of visits during the intervention period. |
| Compared to Usual Care | Millard *et al. ** ^44^ | Identified more out-of-control asthma events. |  | Increased rate of regimen changes for patients with out-of-control asthma. |
|  | Mukherjee *et al. ** ^36^ |  |  | There was a significant reduction in prednisolone prescriptions in the study practice. Other outcomes could not be reported due to small number suppression. |
| Compared to Before Intervention | PAAF ^28^ |  | The PAAF was only used in 12/143 (8%) patients. | There were modest increases in many asthma management processes after the PAAF was introduced, including requesting pulmonary function tests, discussing smoking cessation, delivery of asthma action plans, inhaler technique assessments, and referrals to specialist asthma services.  Fewer patients had poorly controlled asthma, recent emergency department visits, recent hospitalisations, systemic steroids in the last year, or suspected adherence problems after PAAF was implemented. There was a significant increase in the proportion of patients with uncontrolled asthma who had been prescribed an ICS. |
|  | e-MEDRESP ^35^ |  | 15/19 recruited physicians used the system during the trial, and they used it for 34% of consultations, but this declined rapidly during the trial. However, the qualitative evaluation was positive. | No improvement to adherence, SABA use, or OCS use. |
|  | eAAP ^33^ |  |  | Decrease in emergency department visits, hospitalisations, oral steroids, and any exacerbations in children, but not in adults. |
|  | Lachman *et al. ** ^38^ |  |  | ICS and ICS-to-SABA prescribing improved, and asthma related emergency department visits, hospitalisations, and total costs reduced. |
|  | STARRS-GM ^32^ |  |  | Mild increases in ICS prescriptions (5%) and spacers provided (9%). Moderate increases in number of patients with action plans (19%). |
|  | Lou *et al. ** ^39^ |  |  | Less urgent care visits, but no change to emergency department visits or inpatient admissions. |

Notes:

* No project name – lead author of sole publication listed

GeoAsma ^47^ not presented due to lack of published results

Table 5: Randomised Controlled Trials Results

| **Project Name** | **Feasibility** | **Uptake and Acceptability** | **Impact** |
| --- | --- | --- | --- |
| The Breathing for Life Trial ^27^ |  |  | No significant change in asthma attack incidence. |
| ARRISA ^46^ |  |  | No significant change in exacerbations overall, but a reduction in hospitalisations and an increase in oral steroid prescriptions. |
| RAACENO ^53,54^ | 98% of children able to complete FeNO test. | In 25% of assessments where the algorithm recommended a step up, and 25% where it recommended a step down, the algorithm recommendation was not followed. | No significant change in rate of asthma attacks requiring oral steroids, or any secondary outcomes. |
| a-GPS ^34^ | Significantly reduced time for reviewing EHRs for asthma management of each participant. |  | No significant difference in emergency department visit frequency or asthma control.  Large (but not quite significant) reduction in associated healthcare costs. |
| ARRISA-UK ^55^ |  |  | No significant change to incidence of asthma-related hospitalisation and A&E attendance or prescriptions of prednisolone. |
| MOXXI ADS ^31^ | 15% of visits were categorised as out-of-control. However, there was a failure for 16% of visits, in which the system did not alert the user as designed. | The physicians accessed the ADS system in 40% of visits for out-of-control asthma (in which the alert was successful), compared to 5% of visits for in-control asthma. | Significant improvement in ICS/SABA ratio. |
| EPIPP ^30^ |  | A majority of intervention practices found the background information and patient-level data to be moderately or very useful, and the majority of practices reported that the feedback was discussed among the practice team on multiple occasions. | There was a small but significant decrease in patients with inappropriate prescribing. |

Notes:

* No project name – lead author of sole publication listed

Results for ARRISA-UK incomplete (only published conference abstracts available at the time of data extraction).

# Appendix A: Search Strategy

| **PICOS Component** | **Keywords** |
| --- | --- |
| Participant | Asthma |
| Intervention | CCDS  CDSS  Clinical decision  Decision support  Decision making  Expert system  Data-driven  Digital  Computer*  Application  Web  Mobile  Online  Electronic  Risk predict*  Risk strat*  Risk score*  Prognos* |
| Study Design | Trial  Intervention  Controlled  Randomized  Effectiveness  Efficacy  Pilot  Feasibility  Acceptability |

**Pubmed**

1. asthma[Title] OR asthma[MeSH]
2. "Clinical Decision-Making"[Mesh] OR "Decision Support Systems, Clinical"[Mesh] OR "Decision Support Techniques"[Mesh]
3. CDSS[Title/Abstract] OR CCDS[Title/Abstract] OR “clinical decision”[Title/Abstract] OR “decision support”[Title/Abstract] OR “decision making”[Title/Abstract] OR “expert system”[Title/Abstract] “data driven”[Title/Abstract] OR digital[Title/Abstract] OR computer*[Title/Abstract]) OR application[Title/Abstract] OR web[Title/Abstract] OR mobile[Title/Abstract] OR online[Title/Abstract] OR electronic[Title/Abstract] OR “risk predict*”[Title/Abstract] OR “risk strat*”[Title/Abstract] OR “risk scor*”[Title/Abstract] OR prognos*[Title/Abstract]
4. "Clinical Trial Protocol"[Publication Type] OR "Clinical Trial "[Publication Type]
5. trial[Title] OR intervention[Title] OR controlled[Title] OR randomised[Title] OR randomized[Title] OR effectiveness[Title] OR efficacy[Title] OR pilot[Title] OR feasibility[Title] OR acceptability[Title]
6. ("2012/01/01"[Date - Publication] : "3000"[Date - Publication])
7. 2 or 3
8. 4 or 5
9. 1 and 7 and 8 and 6

**the Cochrane central register of controlled trials (CENTRAL)**

1. (asthma):ti or [mh Asthma]
2. [mh "Clinical Decision-Making"] or [mh "Decision Support Systems, Clinical"] or [mh "Decision Support Techniques"]
3. (CDSS):ti,ab,kw or (CCDS):ti,ab,kw or (clinical decision):ti,ab,kw or (decision support):ti,ab,kw or (decision making):ti,ab,kw or (expert system):ti,ab,kw or (data driven):ti,ab,kw or (digital):ti,ab,kw or (data-driven):ti,ab,kw or (computer*):ti,ab,kw or (application):ti,ab,kw or (web):ti,ab,kw or (mobile):ti,ab,kw or (online):ti,ab,kw or (electronic):ti,ab,kw or (risk predict*):ti,ab,kw or (risk strat*):ti,ab,kw or (risk scor*):ti,ab,kw or (prognos*):ti,ab,kw
4. #2 or #3
5. #1 and #4

Filter: (“Cochrane Protocols” or “Trials”) and Published on or after 01/01/2012

**the Health Technology Assessment (HTA) Database**

1. [(asthma)[Title] OR (asthma)[mh]](https://database.inahta.org/search?terms=%28asthma%29%5BTitle%5D%20OR%20%28asthma%29%5Bmh%5D)
2. (Clinical Decision-Making)[mh] OR (Decision Support Systems, Clinical)[mh] OR (Decision Support Techniques)[mh]
3. [CDSS[Title/Abstract] OR CCDS[Title/Abstract] OR "clinical decision"[Title/Abstract] OR "decision support"[Title/Abstract] OR "decision making"[Title/Abstract] OR "expert system"[Title/Abstract] "data driven"[Title/Abstract] OR "data-driven"[Title/Abstract] OR digital[Title/Abstract] OR computer*[Title/Abstract]) OR application[Title/Abstract] OR web[Title/Abstract] OR mobile[Title/Abstract] OR online[Title/Abstract] OR electronic[Title/Abstract] OR "risk predict*"[Title/Abstract] OR "risk strat*"[Title/Abstract] OR "risk scor*"[Title/Abstract] OR prognos*[Title/Abstract]](https://database.inahta.org/search?terms=CDSS%5BTitle%2FAbstract%5D%20OR%20CCDS%5BTitle%2FAbstract%5D%20OR%20%22clinical%20decision%22%5BTitle%2FAbstract%5D%20OR%20%22decision%20support%22%5BTitle%2FAbstract%5D%20OR%20%22decision%20making%22%5BTitle%2FAbstract%5D%20OR%20%22expert%20system%22%5BTitle%2FAbstract%5D%20%22data%20driven%22%5BTitle%2FAbstract%5D%20OR%20%22data-driven%22%5BTitle%2FAbstract%5D%20OR%20digital%5BTitle%2FAbstract%5D%20OR%20computer%2A%5BTitle%2FAbstract%5D%29%20OR%20application%5BTitle%2FAbstract%5D%20OR%20web%5BTitle%2FAbstract%5D%20OR%20mobile%5BTitle%2FAbstract%5D%20OR%20online%5BTitle%2FAbstract%5D%20OR%20electronic%5BTitle%2FAbstract%5D%20OR%20%22risk%20predict%2A%22%5BTitle%2FAbstract%5D%20OR%20%22risk%20strat%2A%22%5BTitle%2FAbstract%5D%20OR%20%22risk%20scor%2A%22%5BTitle%2FAbstract%5D%20OR%20prognos%2A%5BTitle%2FAbstract%5D)
4. 2 or 3
5. 1 and 4

**Embase**

1. Asthma.ti OR exp asthma/
2. Exp Clinical Decision-Making/ OR exp Decision Support Systems, Clinical/ OR exp Decision Support Techniques/
3. (CDSS or CCDS or "clinical decision" or “decision support” or “decision making” or “expert system” or “data driven” or “data-driven” or digital or computer* or web or mobile or online or electronic or “risk predict*” or “risk strat*” or “risk predict*” or “risk scor*” or prognos*).ab,kf,ti.
4. (trial or intervention or controlled or randomised or randomized or effectiveness or efficacy or pilot or feasibility or acceptability or “clinical trial”).ab,kf,ti
5. 2 or 3
6. 5 and 1 and 4

Limit to Published on or after 01/01/2012

**ISRCTN Clinical Trials Registry**

1. Interventions: Decision & Condition: Asthma
2. Interventions: Expert & Condition: Asthma
3. Interventions: Data & Condition: Asthma
4. Interventions: Risk & Condition: Asthma
5. Interventions: Prognostic & Condition: Asthma
6. Interventions: Tool & Condition: Asthma
7. Interventions: System & Condition: Asthma
8. Interventions: Software & Condition: Asthma

# Appendix B: PRISMA Study Flow Diagram

# Appendix C: Data Extraction Sheet

| Study Title |  |
| --- | --- |
| Lead Authors |  |
| Publication Year |  |
| Clinical trial registration |  |
| Project ID |  |
| Country |  |
| Intervention |  |
| Comparator |  |
| What was the proposed mechanism of action for the intervention?  (Select All Applicable) | 1. Improve medication adherence (including inhaler technique) 2. Review or replace pharmacological interventions 3. Adjust modifiable risk factors (such as weight, vaccinations, smoking and more) 4. Increase or instigate self-management 5. Improve reaction time to emergency interventions 6. Other. |
| What was the format or platform of the system? | 1. Software integrated into EHR system 2. Software not integrated into EHR system 3. Mobile device application 4. Web application 5. Other |
| What informed the decision system? | 1. Previously constructed data-driven prediction model 2. Bespoke data-driven prediction model 3. Previously written best practice guidelines 4. Bespoke best practice guidelines 5. Other |
| Was the intervention co-designed with clinicians? |  |
| Was the intervention co-designed with patients? |  |
| Where was the intervention deployed? | 1. Primary Care 2. Secondary Care: Respiratory Specialist 3. Secondary Care: Accident and Emergency 4. Secondary Care: Inpatient Department 5. Other |
| What was the study design? | 1. Randomized Controlled Trial 2. Cluster Randomized Controlled Trial 3. Feasibility or Pilot Study 4. Other |
| Who was the audience of the intervention? | 1. General medical practitioner 2. Specialist respiratory clinician 3. General medical practitioner and Patient 4. Specialist respiratory clinician and Patient |
| If patients were one of the audience groups, what age range? | 1. Children 2. Adolescents 3. Children and Adolescents 4. Adolescents and Adults 5. Adults (including the elderly) 6. The Elderly 7. All Ages 8. Other |
| What was the system output?  (Select All Applicable) | 1. Probability of binary outcome 2. Classifications (such as 'high risk') 3. Recommendations from list of options 4. Risk Factors 5. Checklist of procedures 6. Graphs and visualisations 7. Other |
| Primary Outcome |  |
| What system feasibility and acceptability outcomes were assessed?  (Select All Applicable) | 1. User satisfaction 2. Consultation Time 3. Other |
| What health care process outcomes were assessed?  (Select All Applicable) | 1. Asthma Action Plans Written 2. Prescriptions issued 3. Regimen changes 4. Other |
| What patient-reported outcomes were assessed?  (Select All Applicable) | 1. Asthma control or symptoms 2. Quality of life 3. Self-reported asthma attacks 4. Other |
| What clinical outcomes were assessed?  (Select All Applicable) | 1. Lung function 2. Reliever use 3. Oral steroid prescriptions 4. Unscheduled care contacts 5. Accident and emergency attendance 6. Composite secondary care crisis event 7. Mortality 8. Other |
| Were economic outcomes assessed? |  |
| What other outcomes were assessed? |  |
| What were the patient inclusion criteria? |  |
| How many patients were randomised in the intervention arm? |  |
| How many patients were randomised in the control arm? |  |
| How many clinicians were randomised in the intervention arm? |  |
| How many clinicians were randomised in the control arm? |  |
| What were the results of the study? |  |

# Appendix D: PRIMSA-ScR Checklist

| **Section** | | **Item** | **Checklist Item** | **Page** |
| --- | --- | --- | --- | --- |
| Title | | 1 | Identify the report as a scoping review | 1 |
| Abstract | | 2 | Provide a structured summary that includes (as applicable) background, objectives, eligibility criteria, sources of evidence, charting methods, results, and conclusions that relate to the review questions and objectives. | 2 |
| Introduction | |  |  |  |
|  | Rationale | 3 | Describe the rationale for the review in the context of what is already known. Explain why the review questions or objectives lend themselves to a scoping review approach. | 4 |
|  | Objectives | 4 | Provide an explicit statement of the questions and objectives being addressed with reference to their key elements (for example, population or participants, concepts, and context) or other relevant key elements used to conceptualize the review questions or objectives. | 5 |
| Methods | |  |  |  |
|  | Protocol and Registration | 5 | Indicate whether a review protocol exists; state if and where it can be accessed (for example, a Web address); and if available, provide registration information, including the registration number. | 5 |
|  | Eligibility Criteria | 6 | Specify characteristics of the sources of evidence used as eligibility criteria (for example, years considered, language, and publication status), and provide a rationale. | 5, Table 1 |
|  | Information Sources | 7 | Describe all information sources in the search (for example, databases with dates of coverage and contact with authors to identify additional sources), as well as the date the most recent search was executed. | 5 |
|  | Search | 8 | Present the full electronic search strategy for at least 1 database, including any limits used, such that it could be repeated. | Appendix A |
|  | Selection of Sources of Evidence | 9 | State the process for selecting sources of evidence (that is, screening and eligibility) included in the scoping review. | 6 |
|  | Data Charting Process | 10 | Describe the methods of charting data from the included sources of evidence (for example, calibrated forms or forms that have been tested by the team before their use, and whether data charting was done independently or in duplicate) and any processes for obtaining and confirming data from investigators. | 6 |
|  | Data Items | 11 | List and define all variables for which data were sought and any assumptions and simplifications made. | Appendix C |
|  | Critical Appraisal of Individual Sources of Evidence | 12 | (Optional):  If done, provide a rationale for conducting a critical appraisal of included sources of evidence; describe the methods used and how this information was used in any data synthesis (if appropriate). | N/A |
|  | Summary Measures | 13 | This item from the original PRISMA is not applicable for scoping reviews because a meta-analysis is not done (that is, summary measures are not relevant). | N/A |
|  | Synthesis of Results | 14 | Describe the methods of handling and summarizing the data that were charted. | 6 |
|  | Risk of Bias Across Studies | 15 | This item from the original PRISMA is not applicable for scoping reviews because the scoping review method is not intended to be used to critically appraise (or appraise the risk of bias of) a cumulative body of evidence. | N/A |
|  | Additional Analyses | 16 | This item from the original PRISMA is not applicable for scoping reviews because additional analyses, including sensitivity or subgroup analyses and meta-regression, are not done. | N/A |
| Results | |  |  |  |
|  | Selection of Sources of Evidence | 17 | Give numbers of sources of evidence screened, assessed for eligibility, and included in the review, with reasons for exclusions at each stage, ideally using a flow diagram. | Appendix B |
|  | Characteristics of Sources of Evidence | 18 | For each source of evidence, present characteristics for which data were charted and provide the citations. | Table 2 |
|  | Critical Appraisal Within Sources of Evidence | 19 | (Optional):  If done, present data on critical appraisal of included sources of evidence (see item 12). | N/A |
|  | Results of Individual Sources of Evidence | 20 | For each included source of evidence, present the relevant data that were charted that relate to the review questions and objectives. | Tables 2 to 5 |
|  | Synthesis of Results | 21 | Summarize or present the charting results as they relate to the review questions and objectives. | 6-8 |
|  | Risk of Bias Across Studies | 22 | This item is not applicable for scoping reviews. See explanation for item 15. | N/A |
|  | Additional Analyses | 23 | This item is not applicable for scoping reviews. See explanation for item 16. | N/A |
| Discussion and Funding | |  |  |  |
|  | Summary of Evidence | 24 | Summarize the main results (including an overview of concepts, themes, and types of evidence available), link to the review questions and objectives, and consider the relevance to key groups. | 9 |
|  | Limitations | 25 | Discuss the limitations of the scoping review process. | 10 |
|  | Conclusions | 26 | Provide a general interpretation of the results with respect to the review questions and objectives, as well as potential implications or next steps. | 9-10 |
|  | Funding | 27 | Describe sources of funding for the included sources of evidence, as well as sources of funding for the scoping review. Describe the role of the funders of the scoping review. | 1 |
